# Soluble E-selectin may predict progression of subclinical atherosclerosis, as measured by coronary artery calcium score and aorta calcium score, in women with systemic lupus erythematosus

**DOI:** 10.1186/ar3955

**Published:** 2012-09-27

**Authors:** A Lertratanakul, P Wu, A Dyer, W Pearce, G Kondos, D Edmundowicz, J Carr, R Ramsey-Goldman

**Affiliations:** 1 Northwestern University, Chicago, IL, USA; 2 University of Illinois Chicago, IL, USA; 3 Temple University, Philadelphia, PA, USA

## Background

Women with systemic lupus erythematosus (SLE) have increased rates of subclinical atherosclerosis and cardiovascular (CV) events [1,2]. Circulating adhesion molecules (CAMs) have been associated with subclinical atherosclerosis in SLE patients [3,4]. We investigated the significance of CAMs in subclinical atherosclerosis progression, as measured by the coronary artery calcium score (CAC) and aorta calcium score (AS) in women with SLE.

## Methods

Baseline data collected include demographics and circulating adhesion molecule levels. SLE factors collected included modified SLICC/ACR-DI Damage Index (SDI) (excluding CV outcomes). CAC and AS were measured by electron beam or multidimensional computed tomography at baseline and at one follow-up visit in the Study of Lupus Vascular and Bone Long-Term Endpoints (SOLVABLE). High-risk CAC and AS were defined as CAC >10 and AS >100, respectively. Progression in CAC and AS at follow-up was defined as CAC >10 or AS >100 and >10% increase from baseline. Univariate regression models of CAC and AS with risk factors were examined, and further adjusted for age. CAMs measured were ICAM-1, VCAM-1, soluble E-selectin (sESEL), and CD40L.

## Results

Imaging at baseline and follow-up were performed on 142 subjects; baseline AS scans were not performed in 36 subjects (Table 1). Adhesion molecule levels (Table 2) and imaging marker progression (Table 3) results are presented. In age-adjusted models, only sESEL was significantly associated with AS and CAC progression (Table 4).

**Table 1:** Baseline demographics

	Age (years)	Disease duration (years)	SLEDAI	SDI	Follow-up time (years)
CAC (*n *= 142)	43.3 ± 9.9	12.0 ± 8.4	3.8 ± 3.5	1.5 ± 1.6	3.25 ± 0.35
AS (*n *= 106)	42.2 ± 9.3	12.0 ± 8.5	4.2 ± 3.6	1.6 ± 1.7	3.26 ± 0.35

Data presented as mean ± SD.

**Table 2:** Baseline circulating adhesion molecules

	CAC (*n *= 142)	AS (*n *= 106)
ICAM-1	279.9 ± 91.34	276.83 ± 85.79
VCAM-1	978.0 ± 352.15	973.69 ± 360.05
sESEL	62.45 ± 28.70	63.81 ± 26.89
CD40 ligand	5,984.11 ± 2,971.26	6,026.31 ± 2,885.9

Data presented as mean ± SD.

**Table 3:** Imaging marker progression

	Low risk at baseline		High risk at baseline			
		
	No progression (%)	With progression (%)	With regression (%)	With progression (%)	No progression (%)	Total number with progression (%)
CAC (*n *= 142)	103 (72.5)	12 (8.5)	2 (1.4)	21 (14.8)	4 (2.8)	33 (23.2)
AS (*n *= 106)	67 (63.2)	12 (11.3)	4 (3.8)	23 (21.7)	0	35 (33.0)

**Table 4:** Adhesion molecules regressed against progression in coronary artery calcium score and aorta calcium score

	CAC progression	AS progression
	
Risk factor	OR^a ^(95% CI)	OR^a ^(95% CI)
ICAM-1	0.97 (0.63 to 1.45)	1.30 (0.81 to 2.13)
VCAM-1	1.30 (0.69 to 2.41)	1.85 (0.94 to 3.75)
sESEL	1.68 (1.03 to 2.80)	1.94 (1.08 to 3.60)
CD40 ligand	1.01 (0.65 to 1.56)	0.91 (0.56 to 1.44)

**^a^**Basis for calculation of OR is 1 SD difference.

## Conclusion

A higher level of sESEL is associated with progression in AS and CAC in women with SLE. While previous studies have shown CAMs association with subclinical atherosclerosis [3,4], these results suggest sESEL may predict progression of subclinical atherosclerosis, as measured by AS and CAC, in women with SLE.
